# Public Opinion Leadership in Nursing Practice: A Rogerian Concept Analysis

**DOI:** 10.1177/15271544211071099

**Published:** 2022-01-18

**Authors:** M. van Wijk, P. C. B. Lalleman, G. G. Cummings, J. Engel

**Affiliations:** 1 8119 HU University of Applied Sciences, Utrecht, The Netherlands; 2 3158 University of Alberta, Edmonton, Canada; 3 3158 Fontys University of Applied Sciences, Eindhoven, The Netherlands

**Keywords:** concept analysis, influence, leadership, policy making, public opinion leadership, quality of care

## Abstract

In the Dutch nursing context, work remains in strengthening the voice of nurses serving as frontline health care providers and board members alike. Conceptual clarity of Public Opinion Leadership (POL) in nursing practice is needed to provide attributes, antecedents and consequences for nurses and nurse leaders so they can contribute in the public debate and policy making processes. Using Rodgers’ method of evolutionary concept analysis and the key words “POL,” “lobbying” and “public affairs,” we searched PubMed (including MEDLINE), CINAHL, PsycINFO and Cochrane Library for articles written in English, published between January 1999 and May 2020, which resulted in a final selection of seven studies. In addition, transcripts of an expert panel discussion regarding POL were analyzed. Attributes of POL are credibility, accessibility, altruism, dynamic networking and sense of systemness. Antecedents are a clinical background, authentic authority, policy and political awareness and strategic skills. The main consequences of POL entail influencing those who are involved in policy making processes, a new generation of public opinion leaders, and the raising of bottom-up political leaders. POL is a relatively new concept for nursing, with increasing interest given the need to ensure quality of care by increasing the use of evidence in clinical practice. POL in nursing practice is defined as the action of influencing public debate regarding policy making processes by maintaining dynamic (social) networks, having a high sense of systemness, and being (clinically) credible, altruistic and accessible to peers and a wide variety of stakeholders.

## Introduction

Internationally and in the Netherlands, nurses and nurse leaders are called to show and strengthen their influence to advance the frontline of patient care and the healthcare public policy arena. Recently, Sundean et al. (2020) cites Adams et al. (2019, p. 398) to underscore the challenges of this call, “Nurses” contributions are often completed while leading quietly from the back. This, however, likely has limiting and possibly a detrimental effect on the health of our population.” Although written for an American audience, this quote resonates in the Dutch nursing context where both frontline nurses and board members remain relatively voiceless on crucial healthcare issues on a local and national level. For example, less than 15% of Dutch hospitals have board members with a nursing background (NOS, 2019).

In Dutch clinical practice settings, a recent surge of shared or professional governance structures promotes the inclusion of nurses’ knowledge, skills, and expertize in organizational decision making (Lalleman et al., 2020) and implementation of evidence-based practice (EBP) (van der Goot et al., 2018; van Schothorst–van Roekel et al., 2021). Other literature would describe such knowledge, skills, and expertize of nurses as *local* opinion leadership (LOL) (Flodgren et al., 2019). However, this type of opinion leadership only encapsulates clinical practice, while missing board services and health policy as crucial areas of governance. Therefore, we used LOL as our anchor and starting point for further development and exploration of *public* opinion leadership (POL) in nursing practice.

## Background

In North America there is a growing literature on nurse leaders’ influence on policy participation (Waddell et al., 2017), governance (Porter-O’Grady, 2017; Sundean et al., 2020), educating nurses in health policy (Ellenbecker et al., 2017; Lewinski & Simmons, 2018; Sundean et al., 2019; Thomas et al., 2020), nursing service on boards of directors (Prybil et al., 2019; Sundean et al., 2017), participation in professional nursing organizations (Gebbie et al., 2000) and policy advocacy work (Chiu et al., 2021). In line with other literature, we defined nurse leaders broadly in order to include both formal and informal roles and positions, in and outside the healthcare organization (see for example Cummings et al., 2018 or Sundean et al., 2021). The emerging literature from the United States has highlighted the premature state of research, practice and professional development in the Netherlands with regards to nurse influence, governance and leadership of frontline nurses as well as more established nurse leaders.

A considerable literature base emphasizes the importance of opinion leaders as facilitators in local knowledge transfer to disseminate and implement EBP at the unit level (LOL) (Cranley et al., 2017; Flodgren et al., 2011; Harvey et al., 2019; Kitson et al., 2021). Despite extensive literature about the concept of LOL, the literature base on the concept of POL remains underdeveloped. The recently published scholarly work of Adams et al. (2019), Sundean et al. (2020) and Waddell et al. (2021) regarding influence, helps to shed some light on the concept of POL. Nevertheless, they do not name, frame or define the concept as such resulting in a lack of conceptual clarity about what POL in nursing practice really means. Therefore, in this paper we aim to define the concept of POL in nursing practice.

## Methods

### Study Design

Rodgers’ evolutionary concept analysis was used to analyze the concept of POL in nursing practice (Rodgers & Knafl, 2000). An evolutionary view was appropriate to clarify this continually changing concept and its underlying linguistic behaviors (Rodgers, 1989, 2000b). Furthermore, the concept investigated needs to be considered in relation to the context in which it is used. A seven-phase process according to Rodgers’ inductive method was used in an iterative way to examine, clarify and challenge the meaning of POL in a nursing context (Table 1).

**Table 1.** Seven Steps of Rodgers’ Evolutionary Concept Analysis ∞.

Phase	Step
A. Initial analysis (*introduction*)	I. Specifying a concepts and its alternate terminologies
	II. Determination and selection of the appropriate scope for data collection
(*methods*)	III. Collection of data for concept analysis
B. Core analysis (*results*)	IV. Identifying surrogate and related terms
	V. Data analysis based on concept's attributes, antecedents, consequences
	VI. Description of a model case to illustrate the concept
C. Further analysis (*discussion*)	VII. Further development of the concept through research

∞ Source: Rodgers and Knafl (2000).

This concept analysis is part of an overarching study design, where we employed participatory action research (PAR) to examine POL in nursing practice. The PAR was aligned with a national nurse opinion leadership program, designed and enrolled by the University of Applied Sciences in Utrecht, The Netherlands, in collaboration with the Dutch Nursing Labor Union (NU’91, 2019). In this study participants of the aforementioned nurse opinion leadership program were approached for eligibility in this concept analysis. The program aimed to educate and motivate nurses in their role as public opinion leaders and thereby give voice to daily work and influence policy-making. The participants in the leadership program studied the concept of POL through collective inquiry, as co-researchers. The leadership program took place between April and October 2019, and consisted of six monthly, face-to-face, interactive sessions between participants and key-note speakers. Participants completed individual assignments during and outside their own practice, and outcomes were subsequently shared and reflected on in the larger group of participants. This iterative and reflective process of data collection led to a deeper understanding of POL.

### Literature Search

A literature search was systematically conducted in PubMed (including MEDLINE), CINAHL, Cochrane Library and PsycINFO, up until May 2020. Search strategies were constructed with the assistance of an information specialist from the library of the University of Applied Sciences Utrecht (See Appendix I).

Possible surrogate terms for POL were identified in advance, including (public) opinion leader*, (public) opinion maker, lobbyist, and (*public) affairs officer*. Additionally, to ensure comprehensiveness that all relevant articles were found, the context of nursing was operationalized by searching with the term *health care*. All search terms were combined using Boolean operators. To illustrate, the following search strategy was conducted in PubMed: ((healthcare*[Title/Abstract] OR “health care”)[Title/Abstract]) AND ((“opinion leader*”[Title/Abstract] OR “public opinion leader*”OR “public opinion maker*”[Title/Abstract] OR “opinion maker*”[Title/Abstract] OR lobbyist*[Title/Abstract] OR “public affairs officer*”[Title/Abstract] OR “public affairs*”)[Title/Abstract]). Applied search restrictions were language (English) and publication date (January 1999 to May 2020). This was to facilitate an overview of modern uses of the concept over time, together with the shift towards social media use and emergence of the internet in the last two decades.

One reviewer (MvW) screened all titles and abstracts of articles identified in the initial search. Two reviewers (JE, PL) respectively screened 50% of the initial search results independently of the first reviewer (MvW). Scientific articles or grey literature were selected for full-text screening if (1) POL was a considerable focus point in the title or abstract of the selected article, (2) POL manifested itself in healthcare settings which aligned with nursing practice, (3) the concept of POL was described in OECD countries, as the concept may differ in low income countries (Huertas-Zurriaga et al., 2021) and (4) a description of surrogate/related terms, antecedents, attributes, consequences or examples of POL was provided. Articles were excluded, if (1) local—or product opinion leadership (i.e., implementation processes, change management) was studied or (2) POL was mentioned, but another specific healthcare issue was the main focus, and subsequently leaders in the field were approached to reflect upon and provide their opinion on the specific healthcare issue.

In addition to the electronic search, reference lists of included articles were manually screened, but did not led to any additional articles.

### Panel Discussion

The first of six interactive sessions of the leadership program included a panel discussion, where preliminary results of the concept analyzes were presented to participants and discussed. This aligned with Rodgers’ (1989, 2000b) evolutionary method for concept analysis, by using *verbalized language* about the concept of POL in nursing practice to provide deeper understanding of the concept and strengthen findings of the literature search.

The panel discussion took place on 2nd of April 2019 in Utrecht, The Netherlands, and included three experts in POL and 21 nurses participating in the leadership program (See Table 2 for their demographic characteristics). The three experts came from a diverse set of backgrounds, i.e. professor in nursing (exp1), nurse/social influencer in nursing (exp2) and entrepreneur/lobbyist in nursing (exp3) (Table 2). No relationship was established prior to the panel discussion, except from limited contact on an online forum.

**Table 2.** Demographic Characteristics of Participants of the Panel Discussion.

Id.	Gender	Age	Sector	Highest educational qualification
Experts (*n* = 3)				
Exp 1	Female	53	Hospital care	PhD
Exp 2	Male	29	Primary care	Vocational level
Exp 3	Male	65	Health care	MSc.
Nurse participants leadership program (*n* = 21)				
R1	Female	53	Primary care	Vocational level
R2	Female	31	Hospital care	Vocational level
R3	Female	34	Hospital care	Bachelor level
R4	Male	*	Hospital care	Bachelor level
R5	Male	54	Hospital care	Vocational level
R6	Female	57	Hospital care	Vocational level
R7	Female	48	Hospital care	Bachelor level
R8	Female	39	Mental care	Bachelor level
R9	Female	38	Mental care	Vocational level
R10	Male	35	Hospital care	Bachelor level
R11	Female	24	Hospital care	Bachelor level
R12	Female	28	Hospital care	Bachelor level
R13	Female	32	Hospital care	Bachelor level
R14	Female	*	Primary care	Bachelor level
R15	Female	34	Hospital care	Vocational level
R16	Female	32	Hospital care	Bachelor level
R17	Male	51	Mental care	Vocational level
R18	Female	29	Hospital care	Bachelor level
R19	Male	37	Hospital care	Bachelor level
R20	Female	51	Hospital care	MSc.
R21	Female	54	Hospital care	Vocational level
Total sample	Gender (*female)* n (%)	Age median (range)	Sector n (%)	Highest educational qualification n (%)
*n* = 24	17 (70.8)	41 (24–57)	Primary care 2 (9.5)	Vocational level 8 (33.3)
			Mental care 3 (14.3)	Bachelor level 13 (54.2)
			Hospital care 16 (76.2)	≥MSc. 3 (12.5)

*Note*. EXP = expert panel member; R = respondent panel member; * = missing data.

The *open fishbowl method* was used to structure the panel discussion; a method frequently used to enhance medium to large group discussions (Flor et al., 2013). The method is compatible with principles of participatory research design, as the distinction between speakers and participants is lessened, and involvement of all participants is enhanced despite the large size of the group.

The 1.5-h panel discussion began with a short presentation of preliminary results from the literature search, and was structured in three rounds of 20 min each, guided by the following topics: (1) “How do you define POL?”, (2) “What factors influence POL?”, and (3) “What consequences arise from POL?” The participants of the leadership program and the three experts were separated into an *inner* circle and *outer* circle. In the inner circle (fishbowl), the three experts had a discussion guided by the three main topics introduced by the moderator (PL, lecturer/senior researcher in qualitative research). Participants in the outer circle were placed in subgroups surrounding the inner circle, they listened to the discussion and were able to participate in the discussion on their own initiative or by invitation of the moderator. In addition, each subgroup was asked to make field notes of any thoughts regarding the three discussion rounds, to ensure all participants had an opportunity to share their perception of POL. The panel discussion ended with a debrief where key points were summarized by the moderator and participants were asked to reflect on. Field notes were taken by one of the researchers (MvW, lecturer/junior researcher), and the discussion was audio-recorded and transcribed verbatim for content analysis.

### Data Extraction and Analysis

Findings from each document of the literature search were organized under the concept clarification phases. This led to a first grouping and description of key attributes, antecedents and consequences, which were critically discussed in a consensus meeting with three authors (MvW, PL and JE) to enhance credibility. Then, analysis of the data from the panel discussion took place. The transcript and field notes from the panel discussion were analyzed according to the inductive procedure of conventional content analysis described by Hsieh and Shannon (2005). This method allows for categories to emerge from the data, instead of data analysis being guided by preconceived categories from the analysis of the literature.

First, the transcript of the panel discussion was read repeatedly by MvW, PL and JE for familiarization with the data. Then, each line of the transcript was read word for word by one author (MvW) to derive codes directly from the text (*open coding*), resulting in an initial coding scheme. Field notes were used to interpret the data more carefully. After this initial coding phase, a consensus meeting took place between MvW, PL and JE to discuss and reconcile differences in coding of the data. Based on this meeting the initial coding list was further refined by adding new codes, or reconstructing existing codes.

Second, the codes from the initial code list were sorted into categories to organize related codes into meaningful clusters and then aligned to phases of concept clarification: attributes, antecedents, consequences. Then, findings from the literature and panel discussion were compared and critically reviewed to check for consistency of findings.

Several measures were taken to enhance credibility and dependability of the data-analysis process. First, Atlas.ti V.8 (Atlas.ti Scientific Software Development Company, GmbH, Berlin, Germany) was used to manage the coding process. Second, several consensus meetings took place (MvW, PL, JE, GC) to reach consensus on interpretation of the qualitative data and illuminate blind spots in the analysis process, third aggregated findings and conclusions were shared with participants on two different occasions as a participant check. This, however, did not lead to any additional information or correction of presented data.

Finally, based on the literature search and panel discussion findings, a definition of POL in nursing practice was developed.

### Ethical Approval and Confidentiality

Ethics approval was obtained from the medical ethical review committee of a large academic hospital in the Netherlands (*reference number* 19-102/C), and the University of Alberta in Canada (Pro00111758). Prior to the panel discussion, participants received an information letter explaining details about the study, and written informed consent for participation and audio-taping of the panel discussion was obtained from all participants. Confidentiality was assured by removing traceable information from the transcript related to individual participants. Data were stored on a protected network server at the research institute, only accessible to the research team.

## Results

**Figure 1.**
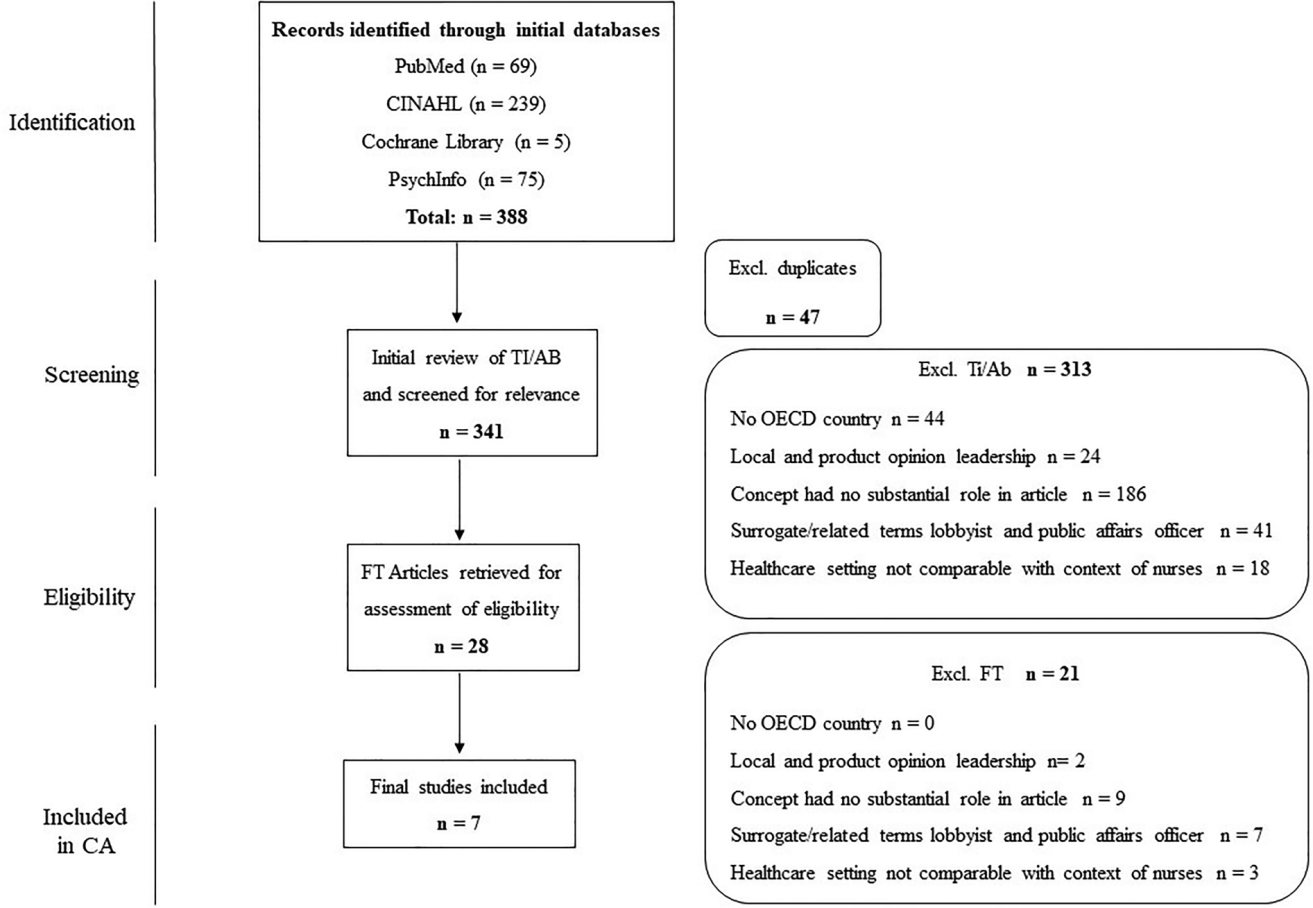
Flowchart concept analysis.

A total of 388 articles were initially retrieved. After duplicates were removed titles/abstracts of articles were screened according to the eligible criteria, resulting in 28 articles for full text screening (see Figure 1). Then, full texts were reviewed, coded (MvW) and collectively discussed by the authors until agreement was reached. This led to seven articles to construct the concept analysis. Figure 1 illustrates the flowchart of the literature search.

In the panel discussion, 21 nurses and three invited experts participated. The majority of participants were women (70.8%) and the mean age was 41 (range 24–57). See Table 2 for all demographic characteristics of the participants.

### Surrogate and Related Terms

Surrogate terms are synonymous and interchangeable with the concept of interest (Rodgers, 2000a). In the selected studies and panel discussion, surrogate terms of the concept POL included “boundary spanner” and (key) “opinion leader”^1^ (Table 3). Both concepts relate to persons who influence policy-making processes within a growing dynamic network. During the initial literature search, “lobbyist” and “public affairs officer” were included as surrogate terms as well. However, conceptual discrepancies of these concepts were apparent in the literature, particularly in relation to persuasive strategies. Lobbying is characterized by more calculated ways of influencing others, while public opinion leaders often use their clinical background as a means to persuade others. Lobbyists and public affairs officers are often commissioned by a company or branch organization rather than operating in their personal interest. Therefore, during the selection process, the authors defined these two concepts as related terms. Related terms refer to “concepts that bear some relationship to the concept of interest but do not seem to share the same set of attributes” (Rodgers, 2000a, p. 92). Numerous related terms were found (Table 3), in which the concept differs from POL due to a distinct interest, objective or position in a social network; e.g., an advice seeker is defined by the quantity of network contacts (Cranley et al., 2019).

**Table 3.** Characteristics of Included Articles and Panel Discussion.

Article/Source	Country of Origin	Study design	Selected key features	Surrogate term	Related term
Panel discussion *n* = 24 participants	Netherlands	Qualitative research	Effect to be credible and acknowledge the role of opinion leader. Intrinsic motivation to influence health policy.	(Opinion) leader	Nurse leader
Cranley et al. (2019)	Canada	Qualitative mixed method—online survey and interviews	Well-connected with broad and deep network linkages, diligent in maintaining connections over career trajectories, and help progress the care and operations of the system well beyond the own organization and its narrower interests.	Opinion leader Boundary spanner	Advice seeker Boundary seeker
Dobrow (2018)	USA	Blog—interview	A respected authority, a thoughtful voice to go to.	Key opinion leader	*
Parish (2001)	UK	News article	Bring everyday healthcare knowledge of nurses to policy making and political influencing processes.	*	Political leader in nursing
Schaeffer and Haebler (2019)	USA	Editorial	Influence the policy and political arena. Bringing a unique perspective on health care	Opinion leader	Advocate Lobbyist
Thompson et al. (2006)	Canada	Concept analysis	Seen as credible, with the ability to persuade others, generally respected authoritative sources of information for a group. Considered knowledgeable, trustworthy, accessible and approachable and willing to share their knowledge.	Opinion leader	Facilitator Informal leader
Waddell et al. (2016)	USA	Case study	Increase nurses’ knowledge of legislative process and health policy issues. Combining policy and clinical (nurse) expertize.	*	Nurse co-lead Active advocates
Welch (2001)	USA	Interview	Experiences, skills and understanding of nursing. Knowing the system of decision making	*	Lobbyist

### Attributes

Attributes represent the primary accomplishment of the concept analysis and together form a definition of opinion leadership in nursing practice (Rodgers, 2000a). From the seven studies analyzed and results of the expert panel discussion, five main attributes were derived: credibility, accessibility, altruism, sense of systemness and dynamic networking (Table 4). Each of the attributes are elaborated below.

**Table 4.** Identification of Attributes, Antecedents and Consequences in Public Opinion Leadership Studies.

	Attributes					Antecedents				Consequences		
*Studies*	Credibility	Accessibility	Altruism	Sense of systemness	Dynamic networking	Clinical background	Authentic authority	Policy and political awareness	Strategical skills	Influence	New generation POL	Bottom up political leaders
Panel discussion	✓		✓		✓	✓	✓		✓	✓	✓	
Cranley et al. (2019)	✓	✓	✓	✓	✓	✓	✓			✓	✓	
Dobrow (2018)	✓	✓	✓			✓	✓			✓		
Parish (2001)					✓	✓		✓	✓	✓		✓
Schaeffer and Haebler (2019)				✓	✓	✓		✓		✓		
Thompson et al. (2006)	✓	✓	✓		✓	✓	✓			✓		
Waddell et al. (2016)				✓	✓	✓		✓	✓	✓	✓	✓
Welch (2001)	✓			✓		✓		✓	✓	✓		

#### Credibility

Four studies and various respondents from the panel discussion identified credibility as a characteristic of POL in nursing practice (Table 4). Credibility refers to public opinion leaders using their often extensive, clinical background to influence policy-making processes (Cranley et al., 2019; Welch, 2001). They are seen as generally respected authoritative sources and their ongoing knowledge and expertize defines them as credible and therefore influential (Dobrow, 2018; Thompson et al., 2006). Secondly, because public opinion leaders are reliable, trustworthy and have clinical expertize, they are a person to whom others go to for advice in more complex situations (Cranley et al., 2019; Thompson et al., 2006). They are endlessly supportive, helpful and are willing to find a solution at any price. Finally, one expert and multiple nurse respondents in the panel discussion mentioned credibility in a way of having in-depth knowledge and enthusiasm which arise from an intrinsic motivation to share, not necessarily in a role of a frontline leader, for example nurse respondent 12:

“… *because you keep a low-profile, i.e. operate in the background, and act based on intrinsic motivation, you are seen as a leader. As soon as the (clinical) content is concerned, then what you have to say is more accepted by colleagues (your supporters).”*

#### Accessibility

Three studies identified accessibility as a key attribute of POL. Accessibility relates to public opinion leaders who are inherently relational and keen to mentor (Cranley et al., 2019; Thompson et al., 2006). Accessibility is often accompanied by terms such as trustworthiness and credibility which refer to the premise that contact with a public opinion leader is easily made (Cranley et al., 2019; Dobrow, 2018; Thompson et al., 2006). Furthermore, accessibility is central to communication structures and information flow (Cranley et al., 2019). Because of social media, such as Twitter, Instagram and LinkedIn, global access is infinite for public opinion leaders. Anyone in healthcare could take the opportunity to share their knowledge and expertize to influence (Dobrow, 2018). Dobrow (2018) exemplifies how social media affects the accessibility of knowledge and influence:

“*Ten years ago, only a few selected people working at topmost organizations—the so-called ‘big names’—would control the direction of healthcare,” … “However, with social media, independent, thoughtful voices got a platform to be heard.”*

Therefore, this social media-era gives a new group of influencers a voice which is easily accessible. Generally, no hierarchical structure underlies these collaboration mechanisms between public opinion leaders and their followers (Dobrow, 2018).

#### Altruism

Altruism was identified as an attribute in three studies and the panel discussion. Altruism refers to the principle and moral practice of doing something for someone else—the opposite of selfishness. Public opinion leaders have the ability to share their expertize on a continuous basis by motivating and providing advice with a strong sense of altruism (Cranley et al., 2019). Nurse respondent 3 in the panel discussion stated:

“*… You can be a role model for your colleagues; collaboratively making sure that patients receive the best possible care. This is my take on leadership.”*

This advisory role emerges from an intrinsic motivation of the public opinion leader towards fellow network members, whereby roles interchange during the process of sharing knowledge and expertize (Cranley et al., 2019; Dobrow, 2018).

“*Opinion leaders are often more ‘cosmopolitan’ than their colleagues, with greater exposure to the bigger picture of advances in their profession beyond the focal advice network, and have displayed a strong sense of ‘altruism’ toward fellow network members.”* (Cranley et al., 2019).

Additionally, altruism involves a certain alliance between persons with the same interests or aims of influencing. Public opinion leaders evaluate their information and interests for fit with local situations and try to obtain group consensus for a greater cause (Thompson et al., 2006). Consequently, their development of joint positions will strengthen each other and may have greater influence in decision-making processes (Dobrow, 2018).

#### Sense of Systemness

Four studies identified sense of systemness as a key attribute. Sense of systemness refers to the multiple dimensions and nuanced perspectives that surround the whole system of healthcare and its political positions and policy-making processes, without the boundaries of a single institution (Waddell et al., 2016; Welch, 2001). Cranley et al. (2019) consider a heightened sense of systemness as a way of sensing responsibility for public opinion leaders to help progress health care, well beyond their own organization and its narrower interests. Public opinion leaders often have an enduring passion and are willing to contribute outside their organization to improve and advance healthcare processes where they possess the clinical expertize (Cranley et al., 2019). Learning from policy makers and political leaders and participating in decision making processes are therefore necessary to gain insight in this “systemness” of healthcare (Schaeffer & Haebler, 2019; Waddell et al., 2016; Welch, 2001).

“*Participation in these organizations provides the nurse co-lead with opportunities to network with many nurse leaders and policy advocates, and lobbyists, to learn about the multiple dimensions and nuanced perspectives that surround policy positions.”* (Waddell et al., 2016).

#### Dynamic Networking

Five studies and the panel discussion identified dynamic networking as a key attribute, which relates to informally well-connected individuals who involve representative stakeholders to help improve care in the complete healthcare system (Cranley et al., 2019; Thompson et al., 2006; Waddell et al., 2016). Public opinion leaders act outside their organizations to fulfill long term relations within the policy arena (Cranley et al., 2019; Waddell et al., 2016). They maintain longstanding connections to broaden and deepen network linkages, in order to create public support and get a decision maker on their side (Cranley et al., 2019; Parish, 2001; Schaeffer & Haebler, 2019). A public opinion leader focuses on peer—and social networks (i.e. social media). The network will be extensive, various and in a constant state of construction and re-construction, in order to continuously add to and refresh a public opinion leader's repertoire of knowledge, expertize and advice (Cranley et al., 2019). Expert respondent 3 in the panel discussion advised:

“*… Start small and stick to what you are good at. … Ask for help and find out who can help you; i.e. a communication department or a full professor. Seek in your network for complementary qualities beyond yours. You do not have to do it on your own.”*

### Antecedents and Consequences

Per Rodgers’ evolutionary method, selected studies and expert panel discussion were reviewed to identify antecedents and consequences of POL in nursing practice. Respectively, these events precede or follow the occurrence of the concept (Rodgers, 2000b; Rodgers & Knafl, 2000). Four antecedents and three consequences submerged from the analysis (Table 4).

#### Antecedents

All studies, as well as the panel discussion, identified *clinical background* as a key antecedent; having a strong nursing background, skills, experiences and an understanding of nursing work to influence policy decisions (Cranley et al., 2019; Dobrow, 2018; Welch, 2001). Public opinion leaders are seen as credible by bringing this everyday knowledge towards the policy tables and therefore contribute to positive change (Parish, 2001; Schaeffer & Haebler, 2019; Waddell et al., 2016). Their clinical knowledge characterizes them as influential and expert (Thompson et al., 2006; Schaeffer & Haebler, 2019). Paradoxically, multiple respondents in the panel discussion noticed a downside of their nursing position in influencing policy making processes by their colleagues, nurse respondent 11 says :

“*Then I am asked: ‘are you still working in healthcare?’. As if I no longer count if I don't. When I work less hours, I already get feedback from my own peers that I belong less. So, if you acknowledge, that you see yourself as an opinion leader, they will cut your head off.”*

Three studies and the panel discussion identified *authentic authority* as a key antecedent. Prior to POL, others recognize them as generally authoritative and respected sources of information for a person or a group (Thompson et al., 2006). The accessibility and willingness of public opinion leaders to share clinical expertize makes them a valuable authentic authority for others (Cranley et al., 2019; Dobrow, 2018). This is recognized by expert respondent 2 in the panel discussion:

“*… gradually I am beginning to realize what the effect of writing my blogs is and how they affect others. […] Opinion leadership can be very small. I don't recognize myself as an influencer, but my blogs affect others and automatically influences others’ perceptions of healthcare related topics.”*

The antecedent, *policy and political awareness,* was reported in four studies. Intrinsic interest in policy work and discussions helps to be recognized as a public opinion leader (Parish, 2001; Waddell et al., 2016). Investing and exploring the political context when joining legislative and policy making processes, develops further understanding of how policy decisions are made (Schaeffer & Haebler, 2019; Waddell et al., 2016; Welch, 2001). This can bridge the gap between the work of legislators, policy-makers and frontline leaders (Schaeffer & Haebler, 2019; Waddell et al., 2016).

Three studies and the panel discussion identified *strategic skills* as an antecedent. A sustained interest in understanding strategic processes of decision-making helps public opinion leaders to learn about multiple dimensions and nuanced perspectives that surround policy decisions (Parish, 2001; Waddell et al., 2016; Welch, 2001). It takes time to initiate lobbying and prepare strategic processes to influence policy makers. Hence, a positive intrinsic motivation for this kind of strategic skill is necessary to keep motivated. Expert respondent 3 in the panel discussion described the following strategic opportunity as an example:

“*It is interesting that there is an academic hospital (in the Netherlands) without a professor of nursing sciences. This in times of scarcity of nurses and the development that the profession is going through at this moment. As an opinion leader you can make use of this situation, by taking the opportunity to publish an opinion paper, and thereby influence the current public opinion and context.”*

#### Consequences

All studies, including the panel discussion, identified *influence* as a consequence of POL. In the context of POL, influence is often based on the premise that people have the ability to persuade others to make important contributions to policy discussions (Cranley et al., 2019; Dobrow, 2018; Parish, 2001; Schaeffer & Haebler, 2019; Thompson et al., 2006; Waddell et al., 2016; Welch, 2001). Public opinion leaders influence public and professional decision-making processes at local, regional and national levels (Parish, 2001). Moreover, they frequently persuade others informally by using substantive arguments, in order to seduce others to broaden or change their perspectives on a decision-making topic (Waddell et al., 2016; Welch, 2001). Nurse respondent 11 cited:

“*Aside from having practical knowledge about the context of patient care, we [nurses] must speak out on different organizational levels where the decisions are being made.”*

To use these substantive arguments from a clinical background perspective makes the public opinion leader a credible influencer (Schaeffer & Haebler, 2019; Waddell et al., 2016). This was recognized by respondents in the panel discussion; expert respondent 1 summarized:

“*… It is essential to work so closely in the field. That is why you [*addressing expert respondent 2, with a clinical background*] earn a lot of credits by your peers. Because it reveals what patients and nurses are dealing with.”*

Two studies and the panel discussion identified *a new generation of public opinion leaders* as a consequence. As a result of POL, existing opinion leaders are good sources for identifying a new generation of public opinion leaders (Cranley et al., 2019). Within the existing interpersonal network, public opinion leaders are trying to impose a change and thereby attract others who are interested in having impact on health policy (Cranley et al., 2019; Waddell et al., 2016). Some respondents in the panel discussion referred to mentorship as a helpful coach to experience, in most cases, a new world of policy knowledge and political strategy. Rather than an external program where formal initiatives dominate; informal contacts emerge in the field where peers are enabled to naturally experience what POL exemplifies. This may lead to a new generation of public opinion leaders, regularly mentored by their colleagues. Nurse respondent 14 stated:

“*We need to help each other as colleagues and be proud of what ‘we’ achieve. … Put someone else in his/her strength and use this for the good cause; a better position of nurses*.”

The consequence, *bottom up political leaders,* was reported in two studies. The foundations for a professional understanding of healthcare policy and political processes appear in the output of POL (Waddell et al., 2016). By being involved in the political arena and identifying the opportunities to influence as a public opinion leader, a continually growing experience could lead to a substantive political position, nationally (Parish, 2001). In the long term, the rise of political leaders from the “bottom up” is essential to bringing clinical knowledge to executive boards and alternative political settings (Parish, 2001; Waddell et al., 2016).

### Ideal Case of POL

An important step in Rodgers (2000a) evolutionary concept analysis is the development of an ideal case (see Box 1). This thoughtful case description of the concept is illustrated in the discipline of nursing, illuminating all the identified attributes and some of the antecedents and consequences.

Box 1. The ideal case.

Holly is a nurse in pediatrics for six years and has advanced education in pediatric oncology. From her early career she shared her ideas and experiences on her social media accounts (Twitter, Instagram and LinkedIn); providing a strong and lively opinion about present topics and concerns in the nursing field. Initially, her thoughtful voice is recognized by her peers. Over the years, her sharp observations combined with humor and witty comments, attracted a broad public on social media. Also, local and national newspapers show interest and recently published one of Holly's opinion articles. Holly's continuous substantive knowledge and curiosity towards policy making decisions leads her to become a member of several healthcare regional committees and boards. All the while she keeps sharing and disseminating these formal discussions through (social) media. She learns to position herself in—frequently complex—policy making cultures, and stands her ground with policy makers and managers. Her credibility and expertize comes from clinical experience and her ability to translate research evidence in her work and opinions. Because of this, Holly is broadly accepted in her role as public opinion leader, and as a result regularly invited to speak on conferences and seminars. Recently, she got an established position as leading expert in the National Nurse Association, which provides her the opportunity to collaborate with the Ministry of Health on several healthcare issues. She has been known to make recommendations that reflect the ideas held by the working nursing staff, i.e. her direct colleagues. Holly is recognized by others as a credible, supportive person who is easily accessible and is always willing to think along with and share her growing dynamic network.

## Discussion

Based on the preceding analysis, the following definition of POL in nursing practice emerged: *POL in nursing practice is the action of influencing public debate regarding policy making processes by maintaining dynamic (social) networks, having a high sense of systemness, and being (clinically) credible, altruistic and accessible to peers and a wide variety of stakeholders*.

This concept analysis provides a clear description of POL which will help to further optimize the effectiveness of public opinion leaders. Moreover, we build on Flodgren et al. (2011) concept of LOL and, with the given description of the concept, address their reviews’ biggest concern, that *“in most of the included studies the role of the opinion leader was not clearly described”* which, according to them, hindered further development and optimization.

When comparing literature focusing on characteristics of LOL versus attributes derived in this concept analyzes of POL, several similarities and differences become apparent. Nurses and nurse leaders are seen as ideal spokespersons for their profession given their authentic authority arising from clinical knowledge and skills. A recent review stresses the importance of authenticity of nurse leaders (Alilyyani et al., 2018). In addition, Lalleman et al. (2016, 2017) underscore having clinical knowledge and skills in leadership as crucial for patient safety practices and patient-centred care. Moreover, having clinical knowledge and skills supports authenticity and also enhances accessibility, which emerged as an attribute of POL. Although accessibility and credibility emerged as important attributes of POL, it is worth noting that being credible as a nurse or nurse leader does not automatically mean you have access to influence on a more macro level. In line with the Woodhull Study on Nursing and the Media (Mason et al., 2018) we see that Dutch nurses and nurse leaders are easily overlooked for advice in national media. However, we believe to see a recent increase in national media outings of nurse and nurse leaders with regards to the COVID-19 pandemic. A Dutch version of the Woodhull Study on Nursing and the Media is therefore advised. Maintaining a dynamic network seems crucial in both LOL as POL in improving quality of care. LOL uses dynamic networking to optimize EBP, by accelerating knowledge transfer and raising awareness (Harvey et al., 2019; Kitson et al., 2021). POL, however, exceeds the local context, and makes use of a dynamic network to gain public support, persuade decision makers and others in making important contributions to policy discussions. There is little insight into such public dynamic networks; therefore, this attribute requires more in-depth inquiry into the social networks of public opinion leaders, for instance through a social network analysis leadership tool (see e.g. Benton, 2015). This way the impact of nurse public opinion leaders in such networks can be better visualized and understood which supports further development and training (see e.g. Marqués-Sánchez et al., 2020).

Our analysis identified a sense of systemness as an important attribute of POL. This is also acknowledged as a key asset of LOL. Kitson et al. (2021) describes that nurse leaders “identified opportunities for local improvement by linking these to wider organizational structures.” In that way, LOL is not entirely focused to the micro context, but also does not exceed the meso (e.g. organizational)level. In contrast with LOL, in which policies and procedures are used to implement EBP in local situations (Kitson et al., 2021), in POL knowledge of hierarchical structures and a system approach are necessary to influence policymaking on a macro (e.g. regional or national) level.

Our results reveal a difference in understanding of POL among nurses who took part in the panel discussion and results from the literature synthesis. Panel participants emphasized that intrinsic motivation is necessary to influence public debate. Whereas in the reviewed studies, multiple attributes (credibility, accessibility, altruism) only implicitly refer to a certain intrinsic motivation. However, there seems to be consensus on public opinion leaders’ altruistic motivations to enhance the position of their profession and ultimately improve quality of care for patients. The importance of altruism could be explained by looking at what Lalleman et al. (2016, 2017) call “a caring disposition of nursing habitus” in which, referring to Levinas, “to take care for the other and not letting the other alone are seen as an indisputable duty” (Levinas & Cohen, 1985 p. 119). Others would say that altruism as a motivating factor in nursing is long overdue and that it is time that the nursing profession examined professional driving forces using more than traditional philosophical frameworks (Haigh, 2010). The latter is interesting because it connotes the urge to develop POL in the context of nursing practice into a strong and effective lobby and strategic public affairs agenda on key nursing and patient issues. In addition, we recognize that it is difficult to actually measure or observe the impact of POL on these issues. Furthermore, literature underlines the importance of developing nurse leadership as stipulated in a recent review of Cummings et al. (2021). Theory based leadership training and mentorship have shown promise in enhancing relational leadership styles among nurse leaders, regardless of training duration.

Several strengths and limitations are worthwhile mentioning. Rodgers’ evolutionary method was used to investigate a concept that is dynamic and still evolving in the practice of nursing. A strength of this study is the involvement of a discussion panel in addition to a thorough literature search in multiple databases, thereby analyzing the concept's use in different data sources, contributing to credibility of study results. In contrast, as individuals who participated in the panel discussion may have been more motivated and engaged about POL than nurses who did not attend this opinion leadership program, transferability of study results is possibly narrowed. For instance, the majority of the panel participants are members of the union board or operate in organizational (nursing) advisory boards.

A first limitation concerns the inclusion criterion regarding only including articles written in English and being OECD countries. The results of this concept analysis therefore only apply for OECD countries and some valuable articles may have been missed that were not published in English. Second, considering that POL is a rather new concept in the healthcare field, some included articles did not explicitly mention POL or provide a clear definition. A recent concept analysis by Sundean et al. (2021) about “influence” shows similarities in attributes with the concept of POL, and could therefore be seen as a related concept. It could be arguable if influence should have been included as a search term. Third, identification of the initial methodological search was done by a single researcher; this may have led to missing relevant articles. However, it could represent an appropriate methodological shortcut in reviews in order to reduce overall workload (Waffenschmidt et al., 2019). Moreover, to avoid as much researcher bias as possible in interpreting the study data, and to enhance objectivity, several consensus meetings among authors were used to critically discuss the coding phases. Also, inductive coding may have contributed to a more comprehensive and credible analysis (Thorne, 2020). Last, while grey literature was not searched extensively, a reasonable amount was included (e.g. Dobrow, 2018; Parish, 2001).

## Conclusion

This concept analysis provides a definition of POL in nursing practice. Identifying the critical attributes and clarifying the definition of POL could intensify further debate regarding involvement and influence of nurses and nurse leaders, not necessarily in their own practice or among their peers, but in the public arena where strategic decisions are made; thereby possibly accelerating this process. Our conceptualization of POL could be used as a framework for developing targeted and tailor-made educational and research programs in which nurses and more established nurse leaders are supported in pursuing their role as public opinion leaders.

## Supplemental Material

sj-docx-1-ppn-10.1177_15271544211071099 - Supplemental material for Public Opinion Leadership in Nursing Practice: A Rogerian Concept Analysis Click here for additional data file. Supplemental material, sj-docx-1-ppn-10.1177_15271544211071099 for Public Opinion Leadership in Nursing Practice: A Rogerian Concept Analysis by M. van Wijk, P. C. B. Lalleman, G. G. Cummings and J. Engel in Policy, Politics, & Nursing Practice

sj-docx-2-ppn-10.1177_15271544211071099 - Supplemental material for Public Opinion Leadership in Nursing Practice: A Rogerian Concept Analysis Click here for additional data file. Supplemental material, sj-docx-2-ppn-10.1177_15271544211071099 for Public Opinion Leadership in Nursing Practice: A Rogerian Concept Analysis by M. van Wijk, P. C. B. Lalleman, G. G. Cummings and J. Engel in Policy, Politics, & Nursing Practice

sj-pdf-3-ppn-10.1177_15271544211071099 - Supplemental material for Public Opinion Leadership in Nursing Practice: A Rogerian Concept Analysis Click here for additional data file. Supplemental material, sj-pdf-3-ppn-10.1177_15271544211071099 for Public Opinion Leadership in Nursing Practice: A Rogerian Concept Analysis by M. van Wijk, P. C. B. Lalleman, G. G. Cummings and J. Engel in Policy, Politics, & Nursing Practice

## Notes

1. None of the included studies combined the term opinion leadership with “public.” Nevertheless, these studies conceptualize opinion leadership in a way that we would describe as “public.”
