# Supplementary material for: Public Opinion Leadership in Nursing Practice: A Rogerian Concept Analysis
Source: Policy Polit Nurs Pract. 2022 Jan 18;23(1):67–79. doi: 10.1177/15271544211071099 (PMC8804936; doi:10.1177/15271544211071099)
Supplement: sj-docx-1-ppn-10.1177_15271544211071099 - Supplemental material for Public Opinion Leadership in Nursing Practice: A Rogerian Concept Analysis [file sj-docx-1-ppn-10.1177_15271544211071099.docx]

**Title: Public opinion leadership in nursing practice: A Rogerian concept analysis**

Running head: Public opinion leadership in nursing practice

van Wijk^a*^, M., Lalleman^a b^, PCB., Cummings^b^, GG., Engel^a^, J.

^a^ HU University of Applied Sciences, Utrecht, The Netherlands

^b^ University of Alberta, Edmonton, Canada

^*^Corresponding author at: Institute of Nursing Studies, HU University of Applied Sciences, Heidelberglaan 7, Postbus 12011, 3501 AA, Utrecht, The Netherlands,
e-mail [marjolein.vanwijk@hu.nl](mailto:marjolein.vanwijk@hu.nl), phone +31641641183

Key words: concept analysis, influence, leadership, policy making, public opinion leadership, quality of care.

**Acknowledgements**

We thank all participants of the panel discussion for their involvement in this study. Furthermore, we thank Kaitlyn Tate, research program manager at the University of Alberta for her contribution as a critical peer during the writing process.
