# Supplementary material for: Public Opinion Leadership in Nursing Practice: A Rogerian Concept Analysis
Source: Policy Polit Nurs Pract. 2022 Jan 18;23(1):67–79. doi: 10.1177/15271544211071099 (PMC8804936; doi:10.1177/15271544211071099)
Supplement: sj-docx-2-ppn-10.1177_15271544211071099 - Supplemental material for Public Opinion Leadership in Nursing Practice: A Rogerian Concept Analysis [file sj-docx-2-ppn-10.1177_15271544211071099.docx]

Appendix I

Search strategy

**CINAHL** (English, January 1999 – May 2020)

| **Search** | **Query** | **Hits** |
| --- | --- | --- |
| #1 | ("opinion leader*" OR “public opinion leader*” OR “public opinion maker*” OR "opinion maker*" OR lobbyist* OR “public affairs officer*” OR “public affairs*”) [tiab] | 1185 |
| #2 | (healthcare* OR “health care”) [tiab] | 321191 |
| #3 | #1 AND #2 | **239** |

**Cochrane Library** (English, January 1999 – May 2020)

| **Search** | **Query** | **Hits** |
| --- | --- | --- |
| #1 | ("opinion leader*" OR “public opinion leader*” OR “public opinion maker*” OR "opinion maker*" OR lobbyist* OR “public affairs officer*” OR “public affairs*”) [tiab] | 7 |
| #2 | healthcare* OR “health care” [tiab] | 906 |
| #3 | #1 AND #2 | **5** |

**PsychINFO** (English, January 1999 – May 2020)

| **Search** | **Query** | **Hits** |
| --- | --- | --- |
| #1 | ("opinion leader*" OR “public opinion leader*” OR “public opinion maker*” OR "opinion maker*" OR lobbyist* OR “public affairs officer*” OR “public affairs*”) [tiab] | 987 |
| #2 | (healthcare* OR “health care”) [tiab] | 330707 |
| #3 | #1 AND #2 | **75** |

**PubMed** (including MEDLINE) (English, January 1999 – May 2020)

| **Search** | **Query** | **Hits** |
| --- | --- | --- |
| #1 | "opinion leader*" OR “public opinion leader*” OR “public opinion maker*” OR "opinion maker*" OR lobbyist* OR “public affairs officer*” OR “public affairs*” [tiab] | 318 |
| #2 | healthcare* OR “health care” [tiab] | 424029 |
| #3 | #1 AND #2 | **69** |
